# Mental Health of Refugees and Torture Survivors: A Critical Review of Prevalence, Predictors, and Integrated Care

**DOI:** 10.3390/ijerph16132309

**Published:** 2019-06-28

**Authors:** Hiba Abu Suhaiban, Lana Ruvolo Grasser, Arash Javanbakht

**Affiliations:** Department of Psychiatry and Behavioral Neurosciences, Wayne State University School of Medicine, Detroit, MI 48201, USA

**Keywords:** torture, refugees, trauma, PTSD, integrated care

## Abstract

Civilian war trauma and torture rank among the most traumatic life experiences; exposure to such experiences is pervasive in nations experiencing both internal and external conflict. This has led to a high volume of refugees resettling throughout the world with mental health needs that primary care physicians may not be screening for and prepared to effectively address. In this article, we review the literature on demographics, predictors, mental health outcomes of torture, and integrated care for the mental health needs of refugees. We searched PubMed and PSYCINFO databases for original research articles on refugees and mental health published in the English language between 2010 and present. Nine percent of 720 adults in conflict areas in Nepal, with predominance of literate married males, met the threshold for Post-Traumatic Stress Disorder (PTSD), 27.5% for depression, and 22.9% for anxiety. While, PTSD rate has been documented as high as 88.3% among torture survivors from Middle East (ME), Central Africa (CA), South Asia (SA), Southeast Europe (SE). Depression was recorded as high as 94.7% among 131 African torture survivors and anxiety as high as 91% among 55 South African torture survivors. Torture severity, post-migration difficulties, and wait time to receive clinical services were significantly associated with higher rate of mental health symptoms. Mental health screening is not a standard component of initial physical exams for refugees, yet these individuals have had high trauma exposure that should inform clinical care. Integrated care models are lacking but would greatly benefit this community to prevent progression to greater severity of mental health symptoms.

## 1. Introduction

Trauma is defined as the exposure to actual or threatened death, serious violence/injury, disaster, or actual or threatened sexual violence via direct experience, witnessing, or learning about the event [1]. Approximately 70% of the general population has been exposed to trauma [2]. For refugees (someone who has been forced to flee his or her country because of persecution, war, or violence [3]) repeated exposure to civilian war trauma and stress of forced migration is common. Traumatic war experiences include detention in concentration camps, separation from family, displacement, witnessing violence, and physical torture. Such events may put one at a greater risk for a wide range of physical and psychological impairment and disability [4,5]. According to the United Nations High Commission on Refugees (UNHCR), there are 65.6 million ‘persons of concern’ worldwide, including 22.5 million refugees and several million asylum seekers (someone who flees his or her own country and seeks sanctuary in another country), internally displaced persons (someone who has been forced to flee his or her home but never crossed an international border), and stateless persons (someone who is not a citizen of any country) [3]. Out of these 22.5 million refugees of war worldwide, up to 35% report being survivors of torture [6,7]. Torture ranks among the most traumatic life experiences, and psychological consequences of torture are often exacerbated by migration (another high-ranking stressful life experience) when an individual flees from harm [8]. Torture is most prevalent in war-torn areas and those where human rights violations are common. The United Nations defines torture as:

“*any act by which severe pain or suffering, whether physical or mental, is intentionally inflicted on a person for such purposes as obtaining from him or a third person information or a confession, punishing him for an act he or a third person has committed or is suspected of having committed, or intimidating or coercing him or a third person, or for any reason based on discrimination of any kind*.”

(United Nations Convention against Torture and Other Cruel UNCAT article)

While physical scars of torture may be more apparent, psychological consequences often go unseen and unaddressed. Exposure to traumatic events may put one at risk for a spectrum of mental health conditions, most commonly acute stress disorder, PTSD, depression, substance use, and increased risk of suicide [9,10]. Depending on the population sampled, between 30% and 80% of refugees screen positive for posttraumatic stress disorder—a far higher rate than that of the general population (8%) [10,11]. Further, it is unknown what resources are made available to refugees within an integrated care model to facilitate the best outcome during resettlement. The National Institute of Mental Health (NIMH) defines integrated care as combining both primary health care and mental health care in one cohesive setting [12]. This model is both practical and essential, as adults with mental illnesses and substance use disorders tend to have higher rates of chronic medical conditions and lower life expectancies than the general population [12]. This integration may be even more necessary for refugees and victims of torture. Fifty-nine percent of refugees and victims of torture report that health professionals, in addition to family members, help support their recovery [13]. It is more than likely that victims of torture experiencing mental health concerns will present to primary care clinics, as comorbid PTSD and depression are associated with elevated rates of physical health conditions [14]. Victims of physical torture will likely present to primary care clinics seeking aid for somatic concerns, however physical torture is correlated with PTSD and insomnia [15], and as such these primary care clinics should also be equipped to address mental health concerns. At the end of a study examining 179 Karen refugees, physicians reported that, over time, they were able to implement a screening tool and address refugees’ mental health complaints more comfortably. They also reported that it was critical to inquire about a patient’s torture history before conducting a physical exam or discussing invasive diagnostic tests [16].

A decade ago, a review was published on association between torture and other traumatic events with mental health outcomes in those exposed to mass conflict and displacement [5]. Due to the large set of heterogenous articles reviewed (161) and high inter-survey variability, nonspecific rates of PTSD and depression were reported ranging from 0% to 99% [5]. Rates of PTSD in refugees do not specifically reflect outcomes for those refugees who have experienced torture. Thus, we conducted an updated critical review of the literature to define types of torture and torture methods, the mental health outcomes of torture, demographic factors related to torture exposure, and predictors of risk and resilience. Predictors of risk and resilience are essential to identify, as this can foster expedited psychiatric exams in primary care settings where time and expertise may be limited; this can also promote best utilization of health care resources to target those individuals who could be most at risk for developing trauma-related disorders such as PTSD, anxiety, and depression. Finally, we also investigated whether any of these reports commented on integrated care for the long-term benefit to refugees experiencing mental health problems.

## 2. Method

**Figure 1:**
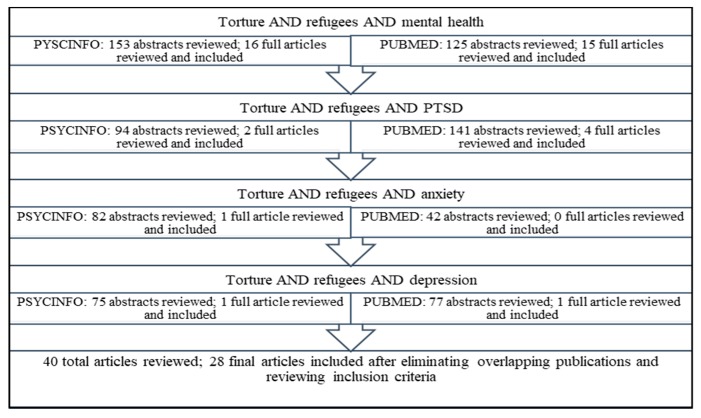
Selection method for research articles to review.

A review of the literature was conducted using the PubMed and PSYCHINFO databases in January 2019 to identify original research publications from 2010 to present, as a major systematic review of torture had previously been conducted on literature through 2009 [2]. Search included the following terms: “torture AND refugee AND mental health,” “torture AND refugee AND PTSD,” “torture AND refugee AND anxiety,” and “torture AND refugee AND depression.” Publications that were not available in English were excluded and publications that provided information regarding prevalence of torture in the sample and/or mental health outcome and/or integrated care were included. Inclusion criteria were adults ages 18+, males and females, and all ethnicities/races. Publications that did not include at least one mental health measure, publications on treatment, or those that included children were excluded. Two authors reviewed the literature using the same combination of key words and inclusion/exclusion criteria. One reviewer searched the PSYCHOIFO database and the other searched PUBMED. Initially, all turned abstracts were reviewed and based on above defined inclusion criteria studies were coded for full article review. Subsequently, both reviewers reviewed and agreed on all included publications. After eliminating overlapping publications, a total of 28 articles meeting the search criteria were identified as below (Figure 1).

## 3. Results

### 3.1. Demographics and Characteristics of Survivors of Torture

Demographic data include age, sex, marital status, education, and religion of refugees (Table 1). Across studies, most individuals are in their 30s and ~60% of individuals are male. Religious persecution is a common reason for torture and forced migration, and Christians and Muslims seem to be equally affected, with 43% of individuals in the present studies reporting as Christians and 43% reporting as Muslim. The majority of tortured adults are married (more than 60%) and in all studies that reported education level, at least one third of respondents had at least primary education or more. Torture is can be classified as either primary or secondary torture. Primary torture is defined as “experiencing torture directly or witnessing the torture of family members” and secondary torture is “reporting torture of a family member, which they did not witness” [16].

Not all studies distinguish between primary and secondary torture; additionally, some use “trauma” and “torture” interchangeably. A summary of trauma exposures, prevalence of torture, type of torture, and any associated demographic characteristics reported is provided in Table 2. Witnessing torture and/or killing of others, physical torture, and sexual torture were the most commonly reported events across studies. While sexual torture does affect both men and women, women are more predominantly affected. Other forms of torture commonly reported include beatings, threats, forced stress positions, sensory stress, psychological manipulation, and separation from family and friend (Table 2). Besides the higher occurrence in women for sexual torture, the present publications did not report any additional demographic characteristics associated with torture methods.

### 3.2. Prevalence of Mental Health Conditions in Refugees and Torture Survivors:

Posttraumatic stress disorder was the predominant mental health outcome measured (Table 3). 

Ibrahim and Hassan (2017) interviewed 91 Syrian Kurdish refugees [18]. Using 2.5 as clinical score on Harvard Trauma Questionnaire (HTQ) (section I, IV, V), 38.46% showed clinically significant posttraumatic stress symptoms [18]. Song et al. (2015) used the PTSD Checklist for Civilians (PCL-C) and Hopkins Symptoms Checklist (HSCL-25) to measure PTSD, anxiety, and depression in 278 tortured refugees from the Middle East. The HSCL is commonly used in refugee populations, and Song et al. (2015) found that 56.9% screened positive for PTSD, 83.8% for depression, and 81.3% for anxiety [38]. Odenat (2012) interviewed 326 adult refugee torture survivors, and 23% were diagnosed with PTSD [17]. Using HSCL-25 (cut-off score = 1.75) for depression and Part 4 HTQ for PTSD (cut-off score = 2.5), Leaman and Gee (2012) reported depression in 94.7% and PTSD in 57.3% of 131 torture survivors from African nations [19]. Using the Diagnostic and Statistical Manual of Mental Disorders, 4th Edition-Text Revision (DSM IV-TR) criteria, 69% of 30 asylees in the United States were diagnosed with PTSD and 69% with depression [29]. In a sample of 720 Adults in Nepal, 9.6% were diagnosed with PTSD using the PCL-C with a threshold score of 50, 27.5% were diagnosed with depression and 22.9% were diagnosed with anxiety using the Beck Depression Inventory (BDI, more than 20), and Beck Anxiety Inventory (BAI, more than 17), respectively [21]. Out of 75 torture survivors resettled in the US, 40.0% had scores above the 2.5 cut-off on HTQ for significant posttraumatic stress symptoms [34]. In a sample of 57 asylum seekers and refugees in Turkey, 55.2% were diagnosed with PTSD and 55.2% with depression using DSM-IV-TR criteria [37]. Bandeira et al. (2010) reported a diagnosis of PTSD in 69% of 55 torture survivors using HTQ and DSM-IV. Using the Hospital Anxiety and Depression Scale (HADS) (score ≥ 11), they also reported depression and anxiety in 74% and 91% of survivors, respectively [13]. Schubert and Punamäki (2011) studied 78 Middle Eastern, Central African, Southern Asian and Southeastern European torture survivors using the Impact of Events Scale-Revised (IES-R) and HSCL-25. A total of 88%, 78.2% and 78.2% of their sample scored above clinical cut-offs for PTSD, depression, and anxiety, respectively [22]. Using the HSCL-25 in a large sample of 1215 refugees, 40.2% were diagnosed with depression (cut-off 1.80) and 31.8% were diagnosed with anxiety (cut-off score = 1.75). Additionally, using HTQ (mean item score of 2.06), 29.9% were diagnosed with PTSD [25]. According to Tamblyn et al. (2011), 48% of 61 torture survivors were diagnosed with PTSD, 45% with depression and 31% with anxiety using the DSM-IV TR criteria [15].

### 3.3. Predictors of Risk and Resilience

Age, sex, torture severity and type, length of time to receive health services, post-migration difficulties, immigration status, social support, marital status, and religion have been reported as potential predictors or protective factors to psychological distress. Song et al. (2015) reported that torture severity, older age, and female sex were significantly associated with increased posttraumatic stress symptoms and depression [38]. Similarly, Ibrahim and Hassan (2017) found a significant positive correlation between posttraumatic stress symptoms and severity of traumatic events. There was no predictive factor of sex and exposure to torture, and no correlation was found between age and the number of traumatic events [18]. Song et al. (2015) studied two groups of torture survivors according to the length of time between arrival in the US and receiving initial clinical services. Participants who received services after one year of resettlement were more likely to have PTSD and/or depression than those who received services immediately following resettlement (within the first year). They also found female sex and religion of Islam (compared to Buddhism and Christianity) to be predictors of high anxiety [38]. Similarly, a large study found that refugees who wait longer to get treatment are at greater risk for depression. They also found that of those who were raped, 62.4% were diagnosed with depression, 79.2% were diagnosed with PTSD, and 46% were diagnosed with both depression and PTSD [26]. In a sample of 134 refugees, Nickerson et al. (2017) reported that women are more likely to have a single psychiatric diagnosis, while men, younger individuals, those with more severe trauma, and post-migration difficulties were more likely to have multiple psychiatric diagnoses [39]. According to Raghavan et al. (2013) improvement of PTSD and depression in refugees was significantly correlated with secure immigration status, and no relationship was found between changes in severity of posttraumatic stress symptoms and sex, age, religion, marital status, or changes in employment [31]. Tufan et al. (2013) found that torture type (experiencing or witnessing torture or death), lack of social support, and loss of a significant other were all predictors of PTSD in asylum seekers and refugees based in Turkey [37]. Odenat (2012) studied 326 adult refugee torture survivors and found repeated torture exposure is associated with greater trauma symptoms and PTSD diagnosis [17]. Similarly, Leaman and Gee (2012) studied 131 torture survivors from African nations and found that sexual torture was significantly associated with posttraumatic stress symptoms, but biological sex was not a significant predictor of PTSD [19]. On the other hand, Luitel et al. (2013) studied 720 adults in conflict areas of Nepal and found that prevalence rates of PTSD, depression, and anxiety were higher among women and older individuals [21]. Chu et al. (2013) examined pre and post-migration factors in 875 survivors of political violence and found that pre-migration experiences such as rape/sexual assault were significantly associated with worse posttraumatic stress outcomes, as were post-migration factors such as measures of financial and legal insecurity [30]. Schubert and Punamäki (2011) examined 78 torture victims from the Middle East, Central Africa, South Asia, and Southeast Europe and found that women had more PTSD and depressive symptoms than men in all cultural groups. Asylum-seeking status was marginally associated with anxiety symptoms only in the Southeastern European group [22]. In a sample of 136 Cambodian refugee adult, Berthold et al. (2014) found that those with comorbid PTSD/depression had higher rate of physical health conditions than those with either PTSD or depression alone [14]. Song et al. (2018) reported anxiety, PTSD, and depression were predicted by female sex, older age, unstable housing, wait time to receive services, and lack of basic resources [27]. Tamblyn et al. (2011) found that physical torture was associated with PTSD and insomnia, while sexual torture was associated with depression, insomnia and somatic symptoms [15]. Finally, in a sample of 497 Iraqi refugees, Willard et al. (2014) reported that those who experienced torture were more likely to have physical and mental symptoms [8].

### 3.4. Integrated Care

We identified only one study published between 2010 and present on integrated care for refugees and torture survivors. Esala et al. (2018) identified the potential benefits and barriers of collaborative care for these populations. This publication showed that meta-analyses based on research in diverse refugees and torture survivors, as well as randomized controlled trials, suggest that collaborative care is effective in improving symptoms of depression and anxiety, increasing treatment satisfaction, medication adherence, severe and persistent mental illness, and key chronic health conditions [40].

## 4. Discussion

The present literature review reflects the experiences of and findings from refugees and survivors of torture from all parts of the globe, including the Middle East, Africa, Europe, Asia, Central America, and South America. Men are more likely to have experienced torture, however a significant portion of women do as well (40%) and women are more likely to experience sexual torture, one of the most commonly reported torture methods, than men. Notably, sexual torture is a specific torture method that significantly predicts more negative mental health, including PTSD, regardless of the sex of the victim. Torture is primarily motivated by political affiliation and religious beliefs, and Christians and Muslims are equally exposed, while Buddhists are also exposed but to a lesser extent. Thus, it is critical not to assume the absence of torture based on sex or religion. In addition, awareness of the motivation for torture as well as the method of torture will be critical for clinicians to guide initial physical health screenings as well as informed, integrative care. During the screening procedure clinicians should be mindful that individuals may have been previously persecuted based on their innate values and characteristics, and as such should prioritize building trust and a safe environment for the evaluation. Especially for women, clinicians should be mindful that sexual violence is a commonly used torture method, and the physical examination should be performed with an understanding of this. Torture is not limited to direct physical harm caused to the individual; torture may be witnessed or psychological in nature—not leaving physical scars visible to the clinician. Therefore, one must not only look for evidence of exposure to harm inflicted on the individual, but also signs of emotional trauma and possible harm to loved ones.

The majority of survivors were married, therefore families of victims of torture may require psychosocial help and support. As torture may be experienced either by the individual him/herself or by witnessing or learning of the torture experience of a loved one, clinicians should be aware that when one family member has been affected, other family members may have been exposed secondarily as well requiring evaluation and potential treatment. There is variability in level of education among survivors, thus it may be prudent to inquire about the educational background during initial screening and provide community resources for education and employment opportunities as post-migration difficulties contribute to psychological distress leading to negative health trajectories.

While most studies of refugees and survivors of torture investigate prevalence of PTSD, it is important to note that anxiety, depression, and other mental health conditions may also be present. Prevalence of PTSD is high among refugee populations in general, and specifically in victims of torture. Most studies reported PTSD rates of 50% or higher among torture survivors, which is significantly greater than civilian (7.8% of general population) and military (30% lifetime prevalence in Vietnam veterans) populations [10,11]. Anxiety and depression were also very highly common; in some studies, up to 90% prevalence among torture victims [41]. PTSD and depression are both highly debilitating conditions [42,43] and are often comorbid. Both on their own and together, PTSD and depression can significantly limit a person’s ability to cope with new life circumstances, such as the stress and limitations caused by forced migration.

Incoming migrants are commonly required to have a routine physical screening at the time of entry to the new country [44]. Given the high level of PTSD, anxiety, and depression across the reviewed studies, screening for mental health consequences of torture and forced migration among refugees in primary care clinics receiving these individuals seems necessary. This will reduce long term mental, physical, and psychosocial impact of these mental disorders in this population, as this risk tends to increase the longer individuals prolong seeking treatment [26,27,39]. Our group has previously shown feasibility of such screening during the initial health screening of both adult and children refugees, although in a research setting [45,46]. Physicians at primary care settings should also consider the influence of culture in the expression of psychological and somatic symptoms [22]. Cultural literacy and awareness are necessary to determine what different populations perceive as a traumatic event [18]. It is of crucial importance to inquire about general understanding of PTSD and cultural variability in the expression of the emotional effects of war trauma [47].

Cultural norms and values form the subjective meaning and perception of trauma. For example, one study using a heterogeneous sample of Hispanic, Non-Hispanic Caucasians, and African American survivors of sudden physical injury found that the Hispanic group reported higher levels of overall posttraumatic distress and symptoms [48]. This may reflect the role of culture on the manifestation of mental health symptoms. Additionally, for some patients who may be reluctant to talk about trauma, the provider can assess the cultural role of family involvement in the treatment and recovery. In addition, being aware of torture motivations among different cultures highlights the need to inquire about torture experiences in a sensitive manner. For example, a study of 278 torture survivors mainly from Iraq, Iran, Eritrea revealed that 38.2% were tortured due to family background, 36.3% due to political beliefs, 30.9% due to religious beliefs and 12.3% due to their ethnicity/race. Early detection and necessary interventions can prevent long term impact of trauma on function, mental, and physical health, and positively promote integration of refugees [14,49,50]. However, it should be noted that traditional forms of care such as Cognitive Behavioral Therapy (CBT) exposure therapy, and pharmacotherapy may face additional challenges in refugee populations, and culturally informed interventions may be required [51]. In all of the reviewed studies, diagnosis was made based on meeting a certain number of symptoms criteria, or passing a threshold of symptoms severity. This is important to consider, because not screening positive, does not mean absence of symptoms, and only shows a lower level of symptoms severity in many of the survivors, which may still need clinical attention. According to this review, the majority of the torture victims were married. As PTSD and depression significantly impair a person’s social and interpersonal function, and anger is a common symptom of PTSD, families of victims of torture often need psychosocial help and support.

This review has a number of limitations. As this was not a systematic review, an assessment on the quality of studies was not performed. We also did not assess the heterogeneity in prevalence and incident of PTSD, anxiety, and depression based on sex, age, place, and culture. Additionally, as this paper does not include any meta-analyses, publication bias has not been accounted for and may influence the findings presented here. Some meta-analyses may access unpublished data for their work, however, we did not access any unpublished data for the present critical review. In another vein, differential chance of publication of large sample studies that have found significant results versus small sample studies that have not found significant effects could have resulted in a bias to this review. Still, this review extends the existing knowledge regarding mental health of refugees and torture survivors.

## 5. Conclusions

Multiple studies show high level of exposure to torture among refugees of all demographics as well as high rates of PTSD, depression, and anxiety among those who were exposed. Despite the high prevalence of trauma-related mental health disorders and the promises of integrated care for refugees and torture survivors, the literature lacks integrated care models implemented with this population. Having mental health care systems established in primary care clinics receiving refugees and torture survivors would allow for better assessment of mental health needs and the provision of early treatment.

## Figures and Tables

**Table 1:** Demographics of refugees and torture survivors.

Study	Sample	Age (Mean)	Gender (Male)	Status (Married)	Education	Religion
Shannon et al. (2016) [16]	179 Karen refugees	35.27	51.4%	62.6%	35.8% completed primary school	-
Odenat (2012) [17]	326 adult refugee torture survivors	38.55	57%	65%	40% completed primary school	-
Ibrahim and Hassan (2017) [18]	91 Syrian Kurdish refugees	29.91	55%	60.4%	5.5% had no formal education	-
Leaman and Gee (2012) [19]	131 African torture survivors	34.12	42%	47.3%	77.1% completed at least high school	Christians 92.4% Muslims 4.6%
McColl et al. (2010) [20]	306 torture survivors from Gaza, Egypt, Mexico, Honduras, South Africa	37.7	56%	42%	30% completed secondary education.	Christians 55% Muslims 38%
Luitel et al. (2013) [21]	720 torture survivors	29.2% ≥ 45 years	51.0%	74.9%	79.2% literate	-
Schubert and Punamäki (2011) [22]	78 torture survivors from Middle East (ME), Central Africa (CA), South Asia (SA), Southeast Europe (SE)	37.60	62.8%	ME: 58.1% CA: 47.8% SA: 69.2% SE: 81.8%	Majority completed secondary school	ME: 58.1% Muslims CA: 91.3% Christians SA: 76.9% Muslims SE: 100% Muslims
Le et al. (2018) [23]	108 refugees in Switzerland	43.2	78.7%	62.1%	41.6% completed secondary	-
Morina et al. (2016) [24]	134 refugees in Switzerland	42	78.4%	58.2%	36.6% completed ≥12 years of education	-
Tinghög et al. (2017) [25]	1215 Syrian refugees resettled in Sweden	32.9% between 30 and 39 years	62.8%	63.5%	40.2% completed 0–9 years	-
NCTTP (2015) [26]	9025 torture survivors	40.18	53%	53%	Mean: 11.8 years of education	Christians 48.0% Muslims 38.5% Buddhist 7.5%
Song et al. (2018) [27]	278 torture survivors mainly from Iraq, Iran, Eritrea	40.31	45.3%	-	36.5% completed ≥13 years	Christians 37.4% Muslims 36% Buddhist 8.6%
Willard et al. (2014) [8]	497 Iraqi refugees in the United States	57.95% between 19 and 64 years	55.5%	-	-	-
Robertson et al. (2016) [28]	449 Somali and Oromo refugee trauma survivors	37.1	52%	32%	50% completed secondary school	-
Asgary et al. (2013) [29]	30 asylee in the United States	31.2	70%	68%	40 % had at least college education	-
Chu et al. (2013) [30]	875 survivors of political violence	34.37	64%	63%	59% completed high school or more	Muslims 38% Christians 31% Buddhist 28%
Raghavan et al. (2013) [31]	172 refugees in the United States	36.9	66.9%	64.5%	-	Muslims 37.2% Christians 33.1% Buddhist 24.4%
Hoffman et al. (2017) [32]	111 Karen refugees	33.7	52%	57%	-	-
Carlsson et al. (2010) [33]	45 (66% Iraqi) refugees	39.2	66.7%	82.2%	-	-
Hooberman et al. (2010) [34]	75 torture survivors in the United States	-	58.7%	54.7%	30.6% completed less than high school	Muslims 42.7% Christians 23.9% Buddhist 14.7%
Kroo and Nagy (2011) [35]	53 Somali refugees	83.0% between 18 and 29 years	83.0%	35.8%	45.3% completed 1–4 elementary educations	-
Berthold et al. (2014) [14]	136 Cambodian refugees	56.5	39%	58.8%	47.8% completed 1–5 years of education	-
Keatley et al. (2015) [36]	85 torture survivors with Traumatic Brain Injury (TBI)	34.18	69.4%	-	49.41% completed post-secondary education	Christians 41.17% Muslims 36.47% Buddhist 15.29%

**Table 2:** Characteristics of torture.

Study	Sample	Trauma (%)	Primary Torture (%)	Secondary Torture (%)	Torture Methods (%)	Demographics
Shannon et al. (2016) [16]	179 Karen refugees	-	27.4	51.4	-	-
Odenat (2012) [17]	326 adult refugee torture survivors	-	-	-	Witnessing (59.5); physical (56.4); sexual (36.5); loss of control of basic life routine (45.4); aggressive environmental control (31.6); formal accusation (30.4)	-
Ibrahim and Hassan (2017) [18]	91 Syrian Kurdish refugees	Three or more events during or after migration (79); forced to flee home country (86.8); witnessed destruction (64.8); confinement due to violence (61.5); exposure to elements (72.6); food/water deprivation (27.5)	-	-	-	-
Leaman and Gee (2012) [19]	131 African torture survivors	-	-	-	Sexual (45); beatings (89); threats to family (48); threats of death (44); verbal abuse (58); food/water deprivation (61)	64% of those who experienced sexual torture were women
McColl et al. (2010) [20]	306 torture survivors from Gaza, Egypt, Mexico, Honduras, South Africa	Ten or more events (10)	76		-	Higher exposure to trauma in men than women
Luitel et al. (2013) [21]	720 torture survivors	Witnessed murders (73); witnessed injury (70); witnessed harassment (68); witnessed destruction of property (60)	-	-	-	-
Schubert and Punamäki (2011) [22]	78 torture survivors from Middle East (ME), Central Africa (CA), South Asia (SA), Southeast Europe (SE)	-	-	-	Death threats and terrorization; witnessing injury/killings; sexual molestations	Sexual torture more common in women; detainment more common in men
Le et al. (2018) [23]	108 refugees in Switzerland	-	-	-	Physical (97.2); forced stress positions (94.4); psychological manipulation (93.5); humiliating treatment (93.5); deprivation of basic needs (91.7); exposure to sensory discomfort (86.1); sexual torture (82.4)	-
Morina et al. (2016) [24]	134 refugees in Switzerland	-	85		Isolation (76.9); imprisonment (76.9); physical assault (75.4); combat (75.4); murder of friend or family member (64.9) forced separation from family (60.4); brainwashing (47.8); disappearing or kidnapping (47)	-
Tinghög et al. (2017) [25]	1215 Syrian refugees resettled in Sweden	Forced separation from friends or family (67.9); loss of significant other (64); witnessed violence or assault (63); sexual violence (7)	31		-	-
NCTTP (2015) [26]	9025 torture survivors	-	-	-	Beating (67.3); threats (67.2); rape (31.1—females; 8.1—male)	Women exposed to rape more than men; age at first torture was 25–44 years
Song et al. (2018) [27]	278 torture survivors mainly from Iraq, Iran, Eritrea	-	-	-	Beating (45.6); wound/maim (8.4); rape/sexual (8.8); forced posture—stretched or hung (11.7); deprivation (17.9); sensory stress (12.4); threats and psychological (56); witnessing (30.8)	Political beliefs (36.3%), family background (38.2%), religious beliefs (30.9%), ethnicity/race (12.3%), and group membership (17.6%) were reasons for torture
Willard et al. (2014) [8]	497 Iraqi refugees in the United States	-	56		Beatings, kidnapping and interrogation, electric shock, rape, witness torture of family member	57.6% of adults; 52.9% of children
Robertson et al. (2016) [28]	449 Somali and Oromo refugee trauma survivors	-	-	-	Physical (71); witness (74); sexual (21)	-
Asgary et al. (2013) [29]	30 asylee in the United States	-	-	-	Blunt force trauma (93)	Political beliefs (63%) and group membership were reasons for torture

**Table 3:** Prevalence of mental health outcomes.

Study	Sample	Measures	PTSD	Depression	Anxiety
Ibrahim and Hassan (2017) [18]	91 Syrian Kurdish refugees	HTQ ^1^ (cut-off 2.5)	35.16% (16 items) 38.46% (45 items)	-	-
Song et al. (2018) [27]	278 torture survivors mainly from Iraq, Iran, Eritrea	PCL ^2^ (45–50 points) HSCL-25 ^3^ (cut-off 1.75)	56.9%	83.8%	81.3%
Odenat (2012) [17]	326 adult refugee torture survivors	Clinician Administered PTSD Scale	23%	-	-
Leaman and Gee (2012) [19]	131 African torture survivors	Part 4 HTQ (cut-off 2.5) HSCL-25 (cut-off 1.75)	57.3%	94.7%	-
Asgary et al. (2013) [29]	30 asylee in the United States	DSM IV-TR	69%	69%	-
Luitel et al. (2013) [21]	720 torture survivors	PCL-C (more than 50 score) BDI ^4^ (more than 20) BAI ^5^ (more than 17)	9.6%	27.5%	22.9%
Hooberman et al. (2010) [34]	75 torture survivors in the United States	HTQ (cut-off 2.5)	40.0%	-	-
Tufan et al. (2013) [37]	57 refugees	DSM-IV-TR	55.2%	55.2%	-
Bandeira et al. (2010) [13]	55 refugees torture survivors	HTQ (cut-off 2.5) HADS ^6^ (more than 11)	69%	74%	91%
Schubert and Punamäki (2011) [22]	78 torture survivors	IES-R ^7^ HSCL-25	88.3%	78.2%	78.2%
Tinghög et al. (2017) [25]	1215 Syrian refugees resettled in Sweden	HTQ (mean item score of 2.06) HSCL-25 (1.80 depression, 1.75 anxiety)	29.9%	40.2%	31.8%
Tamblyn et al. (2011) [15]	61 torture survivors	DSM-IV TR	48%	45%	31%

HTQ ^1^: Harvard Trauma Questionnaire, PCL ^2^: PTSD Checklist, HSCL-25 ^3^: Hopkins Symptoms Checklist, BDI ^4^: Beck Depression Inventory. BAI ^5^: Beck Anxiety Inventory, HADS ^6^: Hospital Anxiety and Depression Scale, IES-R^7^: Impact of Events Scale-Revised.

## References

[1] American Psychiatric Association (2013). Diagnostic and Statistical Manual of Mental Disorders: Diagnostic and Statistical Manual of Mental Disorders.

[2] Kessler R.C., Ustun T.B. (2008). The WHO Mental Health Surveys. Global Perspectives on the Epidemiology of Mental Disorders.

[3] United Nations Convention Against Torture, Article 1.1. http://www.unhcr.org/protection/migration/49e479d10/convention-against-torture-other-cruel-inhuman-degrading-treatment-punishment.html.

[4] Dahl S., Mutapcic A., Schei B. (1998). Traumatic events and predictive factors for posttraumatic symptoms in displaced Bosnian women in a war zone. J. Trauma. Stress.

[5] Steel Z., Chey T., Silove D., Marnane C., Bryant R.A., Van Ommeren M. (2009). Association of torture and other potentially traumatic events with mental health outcomes among populations exposed to mass conflict and displacement: A systematic review and meta-analysis. JAMA.

[6] Campbell T.A. (2007). Psychological assessment, diagnosis, and treatment of torture survivors: A review. Clin. Psychol. Rev..

[7] Dgani-Ratsaby A. (2011). The Effects of Cultural and Familial Factors on Severity of Trauma and Treatment Outcome among a Multicultural Population of Refugee Survivors of Torture. Ph.D. Thesis.

[8] Willard C.L., Rabin M., Lawless M. (2014). The prevalence of torture and associated symptoms in United States Iraqi refugees. J. Immigr. Minor. Health.

[9] Breslau N. (2002). Epidemiologic studies of trauma, posttraumatic stress disorder, and other psychiatric disorders. Can. J. Psychiatry.

[10] Kessler R.C., Sonnega A., Bromet E., Hughes M., Nelson C.B. (1995). Posttraumatic stress disorder in the National Comorbidity Survey. Arch. Gen. Psychiatry.

[11] Weiss D.S., Marmar C.R., Schlenger W.E., Fairbank J.A., Kathleen Jordan B., Hough R.L., Kulka R.A. (1992). The prevalence of lifetime and partial post-traumatic stress disorder in Vietnam theater veterans. J. Trauma. Stress.

[12] (2017). Integrated Care. https://www.nimh.nih.gov/health/topics/integrated-care/index.shtml.

[13] Bandeira M., Higson-Smith C., Bantjes M., Polatin P. (2010). The land of milk and honey: A picture of refugee torture survivors presenting for treatment in a South African trauma centre. Torture J..

[14] Berthold S.M., Kong S., Mollica R.F., Kuoch T., Scully M., Franke T. (2014). Comorbid mental and physical health and health access in Cambodian refugees in the US. J. Community Health.

[15] Tamblyn J.M., Calderon A.J., Combs S., O’Brien M.M. (2011). Patients from abroad becoming patients in everyday practice: Torture survivors in primary care. J. Immigr. Minor. Health.

[16] Shannon P.J., Vinson G.A., Wieling E., Cook T., Letts J. (2015). Torture, war trauma, and mental health symptoms of newly arrived Karen refugees. J. Loss Trauma.

[17] Odenat L. (2012). ‘Means of Survival’as Moderator of the Relationship between Cumulative Torture Experiences and Posttraumatic Stress Disorder among Refugees. Ph.D. Thesis.

[18] Ibrahim H., Hassan C.Q. (2017). Post-traumatic stress disorder symptoms resulting from torture and other traumatic events among Syrian Kurdish refugees in Kurdistan Region, Iraq. Front. Psychol..

[19] Leaman S.C., Gee C.B. (2012). Religious coping and risk factors for psychological distress among African torture survivors. Psychol. Trauma Theory Res. Pract. Policy.

[20] McColl H., Higson-Smith C., Gjerding S., Omar M.H., Rahman B.A., Hamed M., El Dawla A.S., Fredericks M., Paulsen N., Shabalala G. (2010). Rehabilitation of torture survivors in five countries: Common themes and challenges. Int. J. Ment. Health Syst..

[21] Luitel N.P., Jordans M.J., Sapkota R.P., Tol W.A., Kohrt B.A., Thapa S.B., Komproe I.H., Sharma B. (2013). Conflict and mental health: A cross-sectional epidemiological study in Nepal. Soc. Psychiatry Psychiatr. Epidemiol..

[22] Schubert C.C., Punamäki R.L. (2011). Mental health among torture survivors: Cultural background, refugee status and gender. Nord. J. Psychiatry.

[23] Le L., Morina N., Schnyder U., Schick M., Bryant R.A., Nickerson A. (2018). The effects of perceived torture controllability on symptom severity of posttraumatic stress, depression and anger in refugees and asylum seekers: A path analysis. Psychiatry Res..

[24] Morina N., Schnyder U., Schick M., Nickerson A., Bryant R.A. (2016). Attachment style and interpersonal trauma in refugees. Aust. N. Z. J. Psychiatry.

[25] Tinghög P., Malm A., Arwidson C., Sigvardsdotter E., Lundin A., Saboonchi F. (2017). Prevalence of mental ill health, traumas and postmigration stress among refugees from Syria resettled in Sweden after 2011: A population-based survey. BMJ Open.

[26] NCTTP (Member Centers of the National Consortium of Torture Treatment Programs) (2015). Descriptive, Inferential, Functional Outcome Data on 9,025 Torture Survivors Over Six Years in the United States. Torture.

[27] Song S.J., Subica A., Kaplan C., Tol W., De Jong J. (2018). Predicting the mental health and functioning of torture survivors. J. Nerv. Ment. Dis..

[28] Robertson C.L., Savik K., Mathiason-Moore M., Mohamed A., Hoffman S. (2016). Modeling psychological functioning in refugees. J. Am. Psychiatr. Nurses Assoc..

[29] Asgary R., Charpentier B., Burnett D.C. (2013). Socio-medical challenges of asylum seekers prior and after coming to the US. J. Immigr. Minor. Health.

[30] Chu T., Keller A.S., Rasmussen A. (2013). Effects of post-migration factors on PTSD outcomes among immigrant survivors of political violence. J. Immigr. Minor. Health.

[31] Raghavan S., Rasmussen A., Rosenfeld B., Keller A.S. (2013). Correlates of symptom reduction in treatment-seeking survivors of torture. Psychol. Trauma Theory Res. Pract. Policy.

[32] Hoffman S.J., Robertson C.L., Shannon P.J., Cook T.L., Letts J., Mathiason M.A. (2017). Physical correlates of torture exposure in Karen refugees. J. Loss Trauma.

[33] Carlsson J.M., Olsen D.R., Kastrup M., Mortensen E.L. (2010). Late mental health changes in tortured refugees in multidisciplinary treatment. J. Nerv. Ment. Dis..

[34] Hooberman J., Rosenfeld B., Rasmussen A., Keller A. (2010). Resilience in trauma-exposed refugees: The moderating effect of coping style on resilience variables. Am. J. Orthopsychiatry.

[35] Kroo A., Nagy H. (2011). Posttraumatic growth among traumatized Somali refugees in Hungary. J. Loss Trauma.

[36] Keatley E., D’Alfonso A., Abeare C., Keller A., Bertelsen N.S. (2015). Health outcomes of traumatic brain injury among refugee survivors of torture. J. Head Trauma Rehabil..

[37] Tufan A.E., Alkin M., Bosgelmez S. (2013). Post-traumatic stress disorder among asylum seekers and refugees in Istanbul may be predicted by torture and loss due to violence. Nord. J. Psychiatry.

[38] Song S.J., Kaplan C., Tol W.A., Subica A., De Jong J. (2015). Psychological distress in torture survivors: Pre-and post-migration risk factors in a US sample. Soc. Psychiatry Psychiatr. Epidemiol..

[39] Nickerson A., Schick M., Schnyder U., Bryant R.A., Morina N. (2017). Comorbidity of Posttraumatic Stress Disorder and Depression in Tortured, Treatment-Seeking Refugees. J. Trauma. Stress.

[40] Esala J.J., Vukovich M.M., Hanbury A., Kashyap S., Joscelyne A. (2018). Collaborative care for refugees and torture survivors: Key findings from the literature. Traumatology.

[41] Carlsson J.M., Mortensen E.L., Kastrup M. (2006). Predictors of mental health and quality of life in male tortured refugees. Nord. J. Psychiatry.

[42] Foa E.B., Rothbaum B.O., Molnar C. (1995). Cognitive-behavioral therapy of PTSD. Neurobiological and Clinical Consequences of Stress: From Normal Adaptation to PTSD.

[43] Greenberg P.E., Leong S.A., Birnbaum H.G., Robinson R.L. (2003). The economic burden of depression with painful symptoms. J. Clin. Psychiatry.

[44] (2012). CDC—Medical Examination—Immigrant and Refugee Health. https://www.cdc.gov/immigrantrefugeehealth/exams/medical-examination.html.

[45] Javanbakht A., Amirsadri A., Suhaiban H.A., Alsaud M.I., Alobaidi Z., Rawi Z., Arfken C.L. (2018). Prevalence of possible mental disorders in syrian refugees resettling in the united states screened at primary care. J. Immigr. Minor. Health.

[46] Javanbakht A., Rosenberg D., Haddad L., Arfken C.L. (2018). Mental health in Syrian refugee children resettling in the United States: War trauma, migration, and the role of parental stress. J. Am. Acad. Child Adolesc. Psychiatry.

[47] Shannon P.J., Wieling E., McCleary J.S., Becher E. (2015). Exploring the mental health effects of political trauma with newly arrived refugees. Qual. Health Res..

[48] Marshall G.N., Schell T.L., Miles J.N. (2009). Ethnic differences in posttraumatic distress: Hispanics’ symptoms differ in kind and degree. J. Consult. Clin. Psychol..

[49] Bisson J.I., Roberts N.P., Andrew M., Cooper R., Lewis C. (2013). Psychological therapies for chronic post-traumatic stress disorder (PTSD) in adults. Cochrane Database Syst. Rev..

[50] Monson C.M., Macdonald A., Vorstenbosch V., Shnaider P., Goldstein E.S., Ferrier-Auerbach A.G., Mocciola K.E. (2012). Changes in social adjustment with cognitive processing therapy: Effects of treatment and association with PTSD symptom change. J. Trauma. Stress.

[51] Grasser L.R., Javanbakht A. (2019). Treatments of Posttraumatic Stress Disorder in Civilian Populations. Curr. Psychiatry Rep..

